# Low-Cost Real-Time PPP/INS Integration for Automated Land Vehicles

**DOI:** 10.3390/s19224896

**Published:** 2019-11-09

**Authors:** Mohamed Elsheikh, Walid Abdelfatah, Aboelmagd Noureldin, Umar Iqbal, Michael Korenberg

**Affiliations:** 1 Electrical and Computer Engineering Department, Queen’s University, Kingston, ON K7L 3N6, Canada; nourelda@queensu.ca (A.N.); korenber@queensu.ca (M.K.); 2 Electronics and Electrical Communication Engineering Department, Tanta University, Tanta 31512, Egypt; 3 Profound Positioning Inc., Calgary, AB T2P 3G3, Canada; wabdelfatah@profoundpositioning.com; 4 Electrical and Computer Engineering Department, Royal Military College of Canada, Kingston, ON K7K 7B4, Canada; 5 Electrical and Computer Engineering Department, Mississippi State University, Starkville, MS 39762, USA; umar@ece.msstate.edu

**Keywords:** PPP, GNSS, low-cost sensors, PPP/INS integration, vehicle navigation, automated vehicles

## Abstract

The last decade has witnessed a growing demand for precise positioning in many applications including car navigation. Navigating automated land vehicles requires at least sub-meter level positioning accuracy with the lowest possible cost. The Global Navigation Satellite System (GNSS) Single-Frequency Precise Point Positioning (SF-PPP) is capable of achieving sub-meter level accuracy in benign GNSS conditions using low-cost GNSS receivers. However, SF-PPP alone cannot be employed for land vehicles due to frequent signal degradation and blockage. In this paper, real-time SF-PPP is integrated with a low-cost consumer-grade Inertial Navigation System (INS) to provide a continuous and precise navigation solution. The PPP accuracy and the applied estimation algorithm contributed to reducing the effects of INS errors. The system was evaluated through two road tests which included open-sky, suburban, momentary outages, and complete GNSS outage conditions. The results showed that the developed PPP/INS system maintained horizontal sub-meter Root Mean Square (RMS) accuracy in open-sky and suburban environments. Moreover, the PPP/INS system could provide a continuous real-time positioning solution within the lane the vehicle is moving in. This lane-level accuracy was preserved even when passing under bridges and overpasses on the road. The developed PPP/INS system is expected to benefit low-cost precise land vehicle navigation applications including level 2 of vehicle automation which comprises services such as lane departure warning and lane-keeping assistance.

## 1. Introduction

The automotive industry is investing lavishly in the research and development of automated vehicles. Various technologies are developed every day to support and advance the vehicle automation process. The Society of Automotive Engineers (SAE) international standard J3016 defines five levels of vehicle automation: 0 (no automation); 1 (driver assistance); 2 (partial automation); 4 (high automation); and 5 (full automation) [1]. Land vehicles, currently available in the market, employ up to level 2 of automation [2]. The driver assistance features of level 2 such as lane-departure warning and lane-keeping assist require a navigation system with accuracy in the sub-meter level. The implementation of such a navigation system with low-cost requirements is not a trivial task.

Global Navigation Satellite System (GNSS) positioning is the core of vehicle navigation systems [3,4,5]. The demand for GNSS precise positioning is increasing, not only in vehicular navigation but in other applications such as agriculture and remote sensing [6,7,8]. To obtain a high-precision low-cost GNSS solution, Single-Frequency (SF) Precise Point Positioning (PPP) was utilized in this work. SF-PPP has faster convergence than Dual-Frequency (DF) PPP and also comes with a lower cost because it is based on SF measurements which can be obtained from low-cost GNSS receivers. These features of SF-PPP have made it a suitable candidate for dynamic positioning applications with a rapidly changing environment [9].

SF-PPP alone cannot provide a continuous navigation solution for land vehicles because of the frequent GNSS signal blockage due to buildings, trees, tunnels, etc. Therefore, SF-PPP must be integrated with other navigation systems such as the Inertial Navigation System (INS). However, to retain the advantage of using a low-cost GNSS solution, low-cost inertial sensors must also be used [10]. The problem with low-cost sensors, which are typically Micro-Electro-Mechanical Systems (MEMS) sensors, is the excessive error growth in position over time. An uncompensated bias in one of the accelerometers leads to an error in the position over a time interval Δt that is proportional to ${\left(\Delta t\right)}^{2}$, while for gyroscopes it is proportional to ${\left(\Delta t\right)}^{3}$ [11].

Over the last decade, many research papers have investigated PPP/INS integration models, benefits, and performance. That research, however, focused on employing the DF-PPP which requires high-cost GNSS receivers. Furthermore, employing low-cost consumer-grade MEMS sensors for real-time PPP/INS integration has not been investigated in the literature so far.

In [12], DF-PPP using multi-GNSS was integrated with an INS and an odometer. The odometer was used as a measurement update to reduce the Inertial Measurement Unit (IMU) drift. The results showed that the integration with INS enhanced the positioning performance. The accuracy was further improved using the odometer update. The final system achieved decimeter-level Root Mean Square (RMS) positioning accuracy using measurements from both the Global Positioning System (GPS) and the Russian Global Navigation Satellite System (GLONASS). Nevertheless, the testing environment was relatively open-sky, and hence simulated GNSS outages were required to test the integration performance. Moreover, Sensonor’s STIM300 (Horten, Norway) [13] was utilized as a low-cost IMU, but it is more appropriately considered as a high-performance MEMS unit due to its cost of approximately US$ 8000 [14].

Reference [15] presents a tightly-integrated DF-PPP/INS system where single-differencing between satellites was applied. The results showed decimeter-level accuracy during simulated GNSS outages with 10 s and 30 s duration. The authors mentioned that they used IMU-CPT from NovAtel (Calgary, AB, Canada) as a low-cost MEMS IMU; nevertheless, IMU-CPT has fiber optic gyros and MEMS accelerometers and is classified as a tactical-grade IMU [16].

One study for the integration of SF-PPP with a low-cost INS was presented in [17]. The authors developed a SF-PPP model augmented with slant ionospheric delay and receiver Differential Code Bias (DCB) constraints. Virtual observation functions for both the slant ionospheric delay and receiver DCBs were added to the measurement model. The GNSS data was collected using a Trimble NetR9 multi-GNSS receiver (high-cost receiver) (Sunnyvale, CA, USA), whereas the IMU data were collected using a MEMS IMU (POS1100, manufactured by Wuhan MP Space Time Technology Company, (Wuhan, China) with $10^{\circ }/hr$ gyro instability. The RMS position errors of the integrated solution were in the decimeter-level. The test was performed in an open-sky environment; therefore, seven simulated GNSS outages were used to test the integration performance. With seven 30 s GNSS outages and using three-GNSS (GPS, BeiDou, and GLONASS), the RMS position errors were below half a meter.

In the research work mentioned above, the PPP corrections were obtained from the final International GNSS Service (IGS) products [18], which are available for post-mission analysis, not real-time. The performance when utilizing low-cost consumer-grade MEMS sensors with a low-cost GNSS receiver for real-time SF-PPP/INS integration has not been explored. Furthermore, testing the performance of the integrated system was based on simulated GNSS outages, not real GNSS outages.

The aim of this work is to develop an integrated real-time PPP/INS system utilizing a low-cost GNSS receiver and a low-cost IMU. This system can provide a continuous and reliable positioning solution for land vehicle navigation with sub-meter level accuracy in open-sky and suburban areas. The initial results of the developed PPP/INS system were presented at the ION GNSS+ 2018 conference, Miami, FL, USA [19]. In this paper, more details are added to the methodology in addition to extended results and discussions. Moreover, we studied the application of our developed system as a navigation solution for level 2 automated vehicles. We demonstrated and analyzed the performance of our system on highways and suburban areas.

The next section describes the methodology adopted in the implemented system. Section 3 describes the experimental work and achieved results, whereas Section 4 provides the discussion of these results. Finally, Section 5 concludes the presented work.

## 2. Methodology

The implemented integration of SF-PPP and INS is performed in the Loosely-Coupled (LC) mode using an Extended Kalman Filter (EKF). The EKF is the most commonly used filter for integrated navigation systems [20,21]. In the LC mode, the final PPP solution is integrated with the INS solution, which allows employing low-cost GNSS receivers that do not provide raw measurements. The three factors that control the LC GNSS/INS integration performance are the quality of the INS measurements, the accuracy of the GNSS solution, and the fusion algorithm. High-end IMUs could be used to maximize the quality of the INS solution, but it comes with a cost overhead. Therefore, MEMS IMUs are used to meet the low-cost requirement. The performance of the developed system relies on utilizing PPP for a high-quality GNSS solution and a robust PPP/INS fusion algorithm.

**Figure 1:**
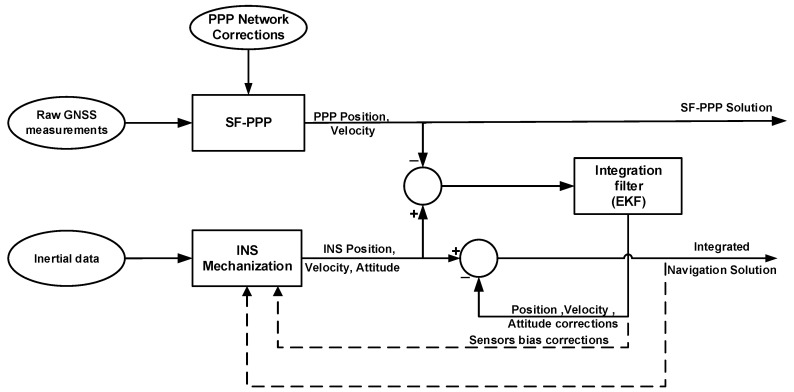
Block diagram of the developed SF-PPP/INS integrated system. The integration is performed in the loosely-coupled mode where the SF-PPP and INS solutions are integrated through an Extended Kalman filter. The dotted lines represent the closed-loop configuration where the estimated errors are fed back to the INS mechanization module.

Figure 1 shows the block diagram of the developed PPP/INS integrated system. The estimated navigation parameters and sensor errors are fed back to the mechanization module in a closed-loop configuration. Thus, the error states are reset every epoch which contributes to preserving the linearity assumption of the KF. The rest of this section gives more details about the implemented SF-PPP, INS mechanization, and the EKF system and measurement models.

### 2.1. Real-Time SF-PPP

SF-PPP relies on the code and phase observations of a single GNSS frequency. The use of multi-constellation contributes to enhancing the availability and accuracy of the PPP solution in the kinematic applications [22]. Therefore, both GPS and GLONASS measurements were adopted in the implemented SF-PPP model. The L1-band frequencies were chosen for both constellations due to their availability in most of the low-cost receivers in the market, especially the C1 code for GPS.

Assuming that the proper correction models have been applied to correct the Sagnac and relativistic effects [23], phase wind-up error [24], receiver antenna phase centers, and site displacement effects [25], the SF observations for GPS and GLONASS can be written as

(1) $$C_{1}^{G}=\rho^{\prime G}+c\left(dt^{r}-dt^{s,G}\right)-B_{C_{1}}^{s,G}+T+I_{1}^{G}+\epsilon_{C_{1}}^{G},$$

(2) $$\Phi_{1}^{G}=\rho^{\prime G}+c\left(dt^{r}-dt^{s,G}\right)+T-I_{1}^{G}+\lambda_{1}^{G}N_{1}+\epsilon_{\Phi_{1}}^{G},$$

(3) $$P_{1}^{R}=\rho^{\prime R}+c\left(dt^{r}-dt^{s,R}\right)+ISB_{G-R}-B_{P_{1}}^{s,R}+T+I_{1}^{R}+\epsilon_{P_{1}}^{R},$$

(4) $$\Phi_{1}^{R}=\rho^{\prime R}+c\left(dt^{r}-dt^{s,R}\right)+ISB_{G-R}+T-I_{1}^{R}+\lambda_{1}^{R}N_{1}+\epsilon_{\Phi_{1}}^{R},$$

where the superscripts *G* and *R* refer to GPS and GLONASS, respectively, $\rho^{\prime }$ is the geometric distance between receiver and satellite, in meters, contaminated by the orbital errors, $dt^{r}$ and $dt^{s}$ are the receiver and satellite clock errors in seconds, and *c* is the speed of light. The term $B^{s}$ represents the satellite DCB, *T* is the tropospheric delay, and *I* is the ionospheric delay, all in meters. The phase integer ambiguity in cycles is denoted by $N_{1}$. The Inter-System Bias (ISB) between GLONASS and GPS clock references is added to GLONASS observations. Finally, ϵ represents the multipath and receiver noise errors.

The most dominant factor that controls real-time PPP accuracy is the availability of precise real-time corrections. Currently, open-access real-time corrections can be obtained either from the IGS Real-time Service (IGS-RTS) or from a Satellite-based Augmentation System (SBAS) [26]. In the implemented SF-PPP, corrections for the satellite orbit and clock errors, ionospheric delays, and code biases are obtained from the Centre National d’Etudes Spatiales (CNES), one of the IGS-RTS analysis centers. CNES products can be received in real-time from the Internet such as the CLK91 stream chosen in this work. Moreover, CNES is the only IGS analysis center transmitting ionospheric corrections so far. The CLK91 stream transmits orbit, clock, and code biases corrections for GPS and GLONASS every 5 s, and transmits ionospheric corrections every 60 s. Finally, the implemented SF-PPP algorithm is designed to use SBAS corrections in case of any interruption to the CLK91 stream.

The total tropospheric delay is modeled a priori using Saastamoinen’s model [27]. After applying all the necessary corrections, the corrected SF-PPP observations are given by

(5) $$C_{1_{corr}}^{G}=\rho +B^{r}+\epsilon_{C_{1}}^{G},$$

(6) $$\Phi_{1_{corr}}^{G}=\rho +B^{r}+\lambda_{1}^{G}N_{1}+\epsilon_{\Phi_{1}}^{G},$$

(7) $$P_{1_{corr}}^{R}=\rho +B^{r}+ISB_{G-R}+\epsilon_{P_{1}}^{R},$$

(8) $$\Phi_{1_{corr}}^{R}=\rho +B^{r}+ISB_{G-R}+\lambda_{1}^{R}N_{1}+\epsilon_{\Phi_{1}}^{R},$$

where ρ is the true geometric range, and $B^{r}=c\left(dt^{r}\right)$ is the receiver clock bias in meters. The unknowns in the previous equations are the three position parameters, the receiver clock bias, the ISB between GLONASS and GPS, and the float ambiguities (one per each satellite). These unknowns were estimated through the SF-PPP navigation filter. The corrected PPP measurements were used as an update to the filter, whereas the prediction of the filter states was based on the stochastic characteristics of each state.

### 2.2. INS Mechanization

**Figure 2:**
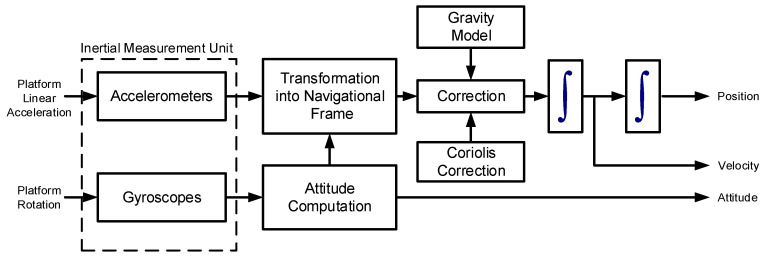
A block diagram of the INS mechanization process [11]. The inputs to the mechanization process are the accelerations and rotations measured by the inertial measurement unit. These measurements are transformed to the navigation frame and integrated to obtain the position, velocity, and attitude parameters.

A standard INS consists of a full IMU system, i.e., it has three orthogonal accelerometers and three orthogonal gyroscopes to measure the accelerations and rotations in all directions in three-dimensional (3D) space. INS mechanization is the process of using the IMU measurements to calculate the position, velocity, and attitude information. The mechanization process starts with a set of initial states and then adds the change in these states at each measurement epoch. Figure 2 shows the general block diagram of the INS mechanization process. The IMU measurements are typically measured in the body (vehicle) frame with respect to the inertial frame; however, the mechanization may be performed in another frame such as the Local-Level Frame (LLF).

The LLF, sometimes called the navigation frame, is a practical choice for vehicular navigation as it provides the position in terms of latitude, longitude, and altitude. The altitude is typically calculated as the ellipsoidal height *h* from the Earth’s ellipsoidal model. However, the orthometric height *H* (above mean sea level) can be obtained if the geoidal height (undulation) *N* was available using the formula h=H+N [28]. In this work, the ellipsoidal height is adopted. The LLF shares the same origin with the vehicle frame, and its axes point to either East, North, and Up (ENU) directions or North, East, and Down (NED) directions. In this work, the ENU directions of the LLF are adopted. More details about the different navigation reference frames can be found in [11].

In the LLF, the position vector $r^{l}$ and the velocity vector $v^{l}$ can be written as

(9) $$r^{l}={\left[\begin{array}{ccc} \varphi & \lambda & h \end{array}\right]}^{T},$$

(10) $$v^{l}={\left[\begin{array}{ccc} v_{e} & v_{n} & v_{u} \end{array}\right]}^{T},$$

where φ is the latitude, λ is the longitude, *h* is the ellipsoidal height, and $v_{e},v_{n},v_{u}$ represent the velocities in east, north and up directions, respectively.

The INS mechanization equations in the continuous-time form are differential equations of the rate of change of the navigation states [11,29]

(11) $$\left[\begin{array}{c} {\dot{r}}^{l}\\ {\dot{v}}^{l}\\ {\dot{R}}_{b}^{l} \end{array}\right]=\left[\begin{array}{c} D^{-1}v^{l}\\ R_{b}^{l}f^{b}-\left(2\Omega_{ie}^{l}+\Omega_{el}^{l}\right)v^{l}+g^{l}\\ R_{b}^{l}\left(\Omega_{ib}^{b}-\Omega_{il}^{b}\right), \end{array}\right]$$

where $R_{b}^{l}$ is the rotation matrix from the body frame to LLF, $f^{b}$ is the vector of specific force measurements from accelerometers in the body frame, and $g^{l}$ is the gravity vector in the LLF. The notation $\Omega_{mn}^{p}$, where the subscripts m,n and the superscript *p* are arbitrary navigation frames, denotes the skew-symmetric matrix form of the angular velocities vector that represent the rotation from n-frame to m-frame measured in p-frame coordinates. The letters *i*, *e*, *l* refer to the inertial frame, Earth-Centered Earth-Fixed (ECEF) frame, and LLF, respectively. $D^{-1}$ is a transformation of the velocity vector $v^{l}$ to geodetic coordinates that uses the meridian radius $R_{M}$ and normal radius $R_{N}$ of the Earth’s ellipsoid and is defined as

(12) $$D^{-1}=\left[\begin{array}{ccc} 0 & \frac{1}{R_{M}+h} & 0\\ \frac{1}{(R_{N}+h)\cos\varphi } & 0 & 0\\ 0 & 0 & 1 \end{array}\right].$$

The mechanization equations in Equation (11) can be intuitively formulated from the block diagram in Figure 2. The position is obtained directly by integrating the velocity, which, in turn, is obtained from integrating the acceleration. The acceleration $f^{b}$ is measured in the body frame with respect to the inertial frame, and hence must be transformed first to the LLF using $R_{b}^{l}$. In addition, the gravity and Coriolis effects must be removed to obtain the motion acceleration. Therefore, the vehicle acceleration ${\dot{v}}^{l}$ has correction terms for the gravity vector $g^{l}$ and Coriolis effects $\left(2\Omega_{ie}^{l}+\Omega_{el}^{l}\right)v^{l}$. These Coriolis effects combine the effect of Earth rotation with respect to the inertial frame and the movement of LLF over Earth’s curvature [11].

The vehicle attitude is represented by the three Euler angles: pitch (*p*), roll (*r*), and azimuth (Az) [29]. The pitch angle describes the rotation around the *x*-axis (lateral direction) of the vehicle frame, whereas the roll angle is the rotation around the *y*-axis (forward direction). The azimuth angle is the rotation around the *z*-axis (up direction) measured clockwise between the vehicle forward direction and the Earth’s north direction. The matrix $R_{b}^{l}$ is given by

(13) Rbl=−cosAzcosr+sinAzsinpsinrsinAzcosp−cosAzsinr−sinAzsinpcosr−sinAzcosr+cosAzsinpsinrcosAzcosp−sinAzsinr−cosAzsinpcosr−cospsinrsinpcospcosr.

The third equation in Equation (11), ${\dot{R}}_{b}^{l}=R_{b}^{l}\left(\Omega_{ib}^{b}-\Omega_{il}^{b}\right)$, together with Equation (13) are needed to calculate the attitude angles. However, the solution to this problem cannot be obtained in closed form and requires numerical integration methods such as the quaternion approach.

### 2.3. System Model

The basic concept of the EKF is that the errors in the system states can be assumed to be linear when the absolute states itself cannot [30]. The system error model consists of fifteen error states that can be grouped into five 3×1 column vectors: position errors $\delta r^{l}$, velocity errors $\delta v^{l}$, attitude errors $\delta \psi^{l}$, accelerometers biases $b_{a}$, and gyroscope biases $b_{g}$. The state vector δX can be described as follows:

(14) $$\delta X_{15\times 1}={\left[\begin{array}{ccccc} \delta r^{l} & \delta v^{l} & \delta \psi^{l} & b_{a} & b_{g} \end{array}\right]}^{T}.$$

The system model can be described in the continuous-time domain using

(15) $$\delta \dot{x}=F\delta X+W,$$

where *F* is the system dynamic coefficient matrix, and W is the process noise vector with covariance matrix *Q*.

For better long-term performance, the implemented system model considered the INS error terms with small values in the *F* matrix. The derivation and components of the dynamic coefficient matrix *F* can be found in [29]. The errors of the accelerometers and gyroscopes were modeled using the first-order Gauss–Markov process.

In the discrete implementation of the EKF, the prediction is based on the state transition matrix ϕ which can be related to *F* using the formula

(16) ϕ≈I+FΔt,

where *I* is the identity matrix and Δt is the time interval between current and previous IMU measurement epochs.

The discrete EKF equations are used to predict the current states and its a priori covariance $P_{k}^{-}$ at epoch *k* based on their values from the previous epoch. The EKF prediction equations in closed-loop configuration can be written as

(17) $${\hat{\delta x}}_{k}^{-}=0,$$

(18) $$P_{k}^{-}=\phi_{k-1}P_{k-1}^{+}\phi_{k-1}^{T}+Q_{k-1},$$

where $P_{k-1}^{+}$ is the posterior state covariance matrix of the previous epoch.

### 2.4. Measurement Model

The measurement model is described by

(19) δZ=HδX+η,

where δZ is the measurement error vector, *H* is the measurement design matrix, and η is the measurement noise with covariance matrix *R*.

The measurement design matrix *R* can be directly taken from the covariance of the PPP solution. However, in the implemented algorithm, the covariance of the PPP solution is passed to a multi-level scaling module based on the other PPP statistics such as the Dilution of Precision (DOP), and the number of visible satellites. This scaling has contributed to a better performance in challenging GNSS environments.

The measurement error vector δZ represents the difference between the values predicted by the INS system model and the update observations. When taking the update from the PPP solution, this difference can be calculated using

(20) $$\delta Z_{PPP}=\left[\begin{array}{c} r_{INS}^{l}-r_{PPP}^{l}\\ v_{INS}^{l}-v_{PPP}^{l} \end{array}\right],$$

where $r_{PPP}^{l}$ and $v_{PPP}^{l}$ are the position and velocity vectors obtained from the PPP solution and represented in the LLF. The design matrix is given by

(21) $$H_{PPP}=\left[\begin{array}{cc} I_{6\times 6} & 0_{6\times 9} \end{array}\right].$$

In the case of a GNSS signal blockage, the update from PPP is not available. Using only the INS solution, especially with low-cost sensors, will lead to a large solution drift. For land vehicles, some constraints can aid the INS during GNSS outages. Two examples of these constraints, which are applied in this work, are the Zero-Velocity Update (ZUPT) and the Nonholonomic Constraints (NHC) [31].

In ZUPT, when the vehicle is detected to be static, all the velocities should be zero. This fact is used to reset the velocity errors and limit the position error growth. ZUPT can be useful in many application not only the car navigation [32]. The measurement error vector and design matrix when using ZUPT are 


(22) $$\delta Z_{ZUPT}=\left[\begin{array}{c} v_{INS}^{l}-0_{3\times 1} \end{array}\right],$$

(23) $$H_{ZUPT}=\left[\begin{array}{ccc} 0_{3\times 3} & I_{3\times 3} & 0_{3\times 9} \end{array}\right].$$

The ZUPT detection module is designed to compare the variance of the forward acceleration against a threshold value. The threshold starts with a predefined value, and then goes through an online detection algorithm to refine this value ongoing.

The NHC in land vehicles are based on the fact that the vehicle does not slip or fly, which means that the vehicle velocity in the lateral and up directions is close to zero. Thus, the measurement vector of the NHC update is represented using the INS velocity in the body frame

(24) $$\delta Z_{NHC}=\left[\begin{array}{c} v_{INS,lateral}^{b}-0\\ v_{INS,up}^{b}-0 \end{array}\right].$$

In [33], the error in the velocity in the body-frame was related to the velocity error in the LLF and the attitude errors by the formula

(25) $$\delta v^{b}=R_{l}^{b}\delta v^{l}-R_{l}^{b}\left(v^{l}\times \right)\delta \psi^{l},$$

where $\left(v^{l}\times \right)$ is the skew-symmetric form of the velocity error vector in the LLF. Following the ENU directions order and assuming the forward motion is in the *y*-direction of the body frame, the design matrix of the NHC update can be written as

(26) $$H_{NHC}=\left[\begin{array}{ccccccc} 0_{1\times 3} & R_{11} & R_{21} & R_{31} & -v_{u}R_{21}+v_{n}R_{31} & v_{u}R_{11}-v_{e}R_{31} & -v_{n}R_{11}+v_{e}R_{21}\\ 0_{1\times 3} & R_{31} & R_{32} & R_{33} & -v_{u}R_{32}+v_{n}R_{33} & v_{u}R_{31}-v_{e}R_{33} & -v_{n}R_{13}+v_{e}R_{32} \end{array}\right],$$

where $R_{ij}$ is the element at row *i* and column *j* of the matrix $R_{l}^{b}$ which is the transpose of the matrix defined in Equation (13).

The EKF update equations are used to update the system states and its posterior covariance matrix $P_{k}^{+}$ as follows:

(27) $$K_{k}=P_{k}^{-}H_{k}^{T}{\left\{H_{k}P_{k}^{-}H_{k}^{T}+R_{k}\right\}}^{-1},$$

(28) $$\delta {\hat{x}}_{k}^{+}=\delta {\hat{x}}_{k}^{-}+K_{k}\left(\delta Z_{k}-H_{k}\delta {\hat{x}}_{k}^{-}\right),$$

(29) $$P_{k}^{+}=\left(I-K_{k}H_{k}\right)P_{k}^{-},$$

where $K_{k}$ is the Kalman gain.

## 3. Experiments and Results

To assess the developed PPP/INS system in real time, two road test trajectories were performed. The first trajectory examines the open-sky and suburban performance, whereas the second trajectory includes more challenging conditions such as high dynamics, overpass bridges, and a complete GNSS outage. This section starts with the experimental setup used in these tests; then, the results obtained from each trajectory are displayed.

### 3.1. Experimental Setup

The test data were collected using a testing van where the GNSS antennas were put on the roof. The remaining equipment was mounted on a flat platform that is firmly attached to the testing vehicle such that the IMU frame is oriented with the vehicle frame to the maximum possible extent.

The SF-GNSS measurements, for GPS and GLONASS, were obtained from the low-cost u-blox EVK-8MT receiver (Thalwil, Switzerland). Moreover, precise satellite orbit and clock corrections every 5 s and ionospheric corrections every 1 min were received in real time from the CLK91 stream of the CNES analysis center. SBAS corrections were also logged by the GNSS receiver to be used in case of any interruption to the CLK91 stream.

In the INS part, LSM6DSL, a low-cost consumer-grade MEMS IMU was utilized [34]. The results are compared to u-blox EVK-M8U Untethered Dead Reckoning (UDR) solution, which is a benchmark in the navigation market for low-cost GNSS/INS applications at the time these tests were performed. The u-blox receiver was configured to use SBAS corrections to get the best-integrated solution for comparison. The reference was obtained from a DGNSS/INS integrated solution where the real-time rover data was collected using the NovAtel SPAN on a ProPak6 system (Calgary, AB, Canada) with IMU-KVH [35] as a tactical-grade IMU. The IGS UCAL station was used as a reference station with a maximum baseline length of 12 km. Furthermore, the reference data were post-processed using NovAtel’s Waypoint Inertial Explorer software.

### 3.2. Road Test Trajectory 1

**Figure 3:**
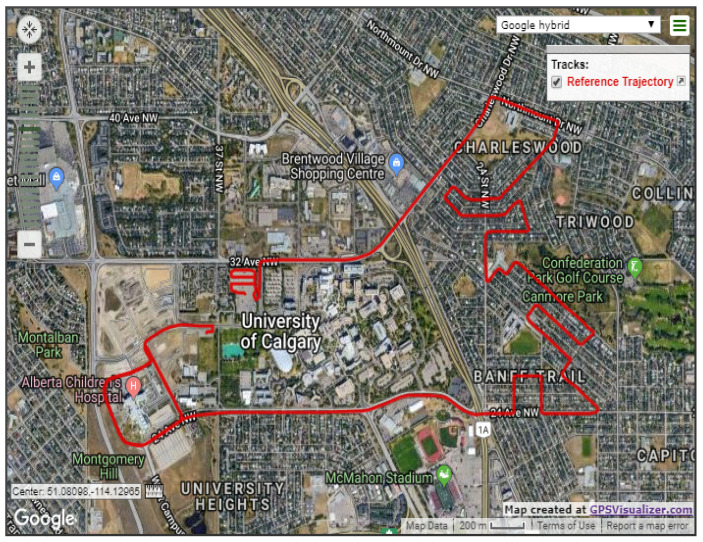
The first test trajectory of SF-PPP/INS integration, Calgary, Alberta, Canada.

The first road test trajectory lasted approximately 35 min in Calgary, Alberta, Canada. Figure 3 shows the trajectory on a Google map; the test started from an open-sky condition at the University of Calgary and near the Alberta Children’s Hospital, and then the car moved towards a residential area. The residential area was a typical suburban environment with community houses and trees on both sides of the road. Finally, the car moved back toward the university.

**Figure 4:**
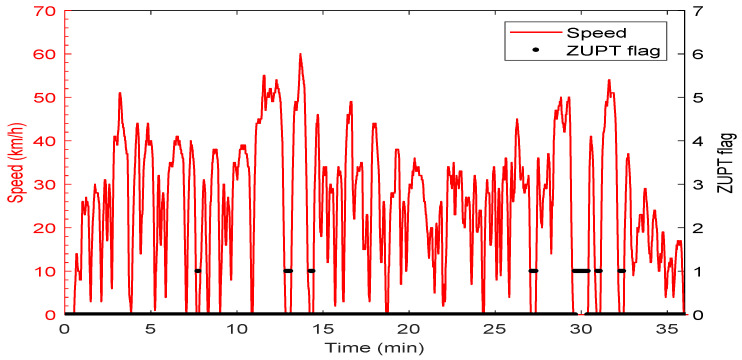
Vehicle speed and Zero-Velocity Update (ZUPT) flag of the first trajectory.

**Figure 5:**
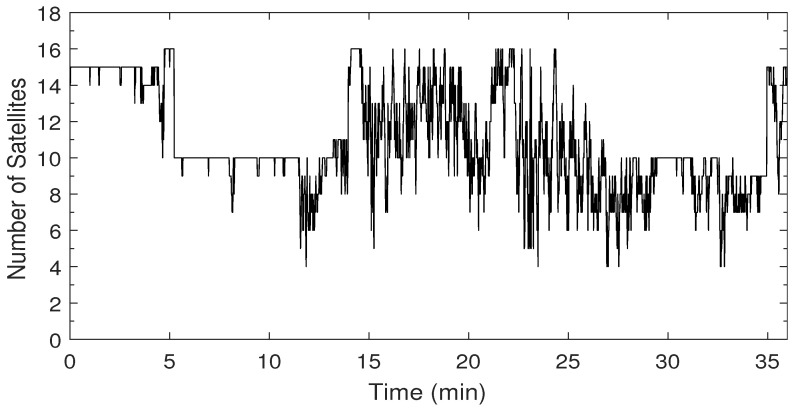
Number of satellites used in the PPP solution of the first trajectory.

The speed profile and the corresponding ZUPT detection flag are shown in Figure 4. The car forward speed, measured by a car odometer, was less than 60 km/h with frequent stops and low-speed periods, which is typical for a suburban area. The ZUPT flag is raised whenever the vehicle is detected to be static. The value of the ZUPT flag in this test indicates the success of the ZUPT detection module with zero false alarms. A few misdetections were found that correspond to vehicle stops that are less than the adequate time needed to declare a stop with high confidence. Nevertheless, as mentioned earlier, the ZUPT update is not used when there is a reliable velocity update from the PPP solution. Figure 5 shows the number of satellites used in the PPP solution versus time. A minimum number of four satellites were seen in this test, whereas the maximum number was 16 thanks to employing both GPS and GLONASS satellites.

**Figure 6:**
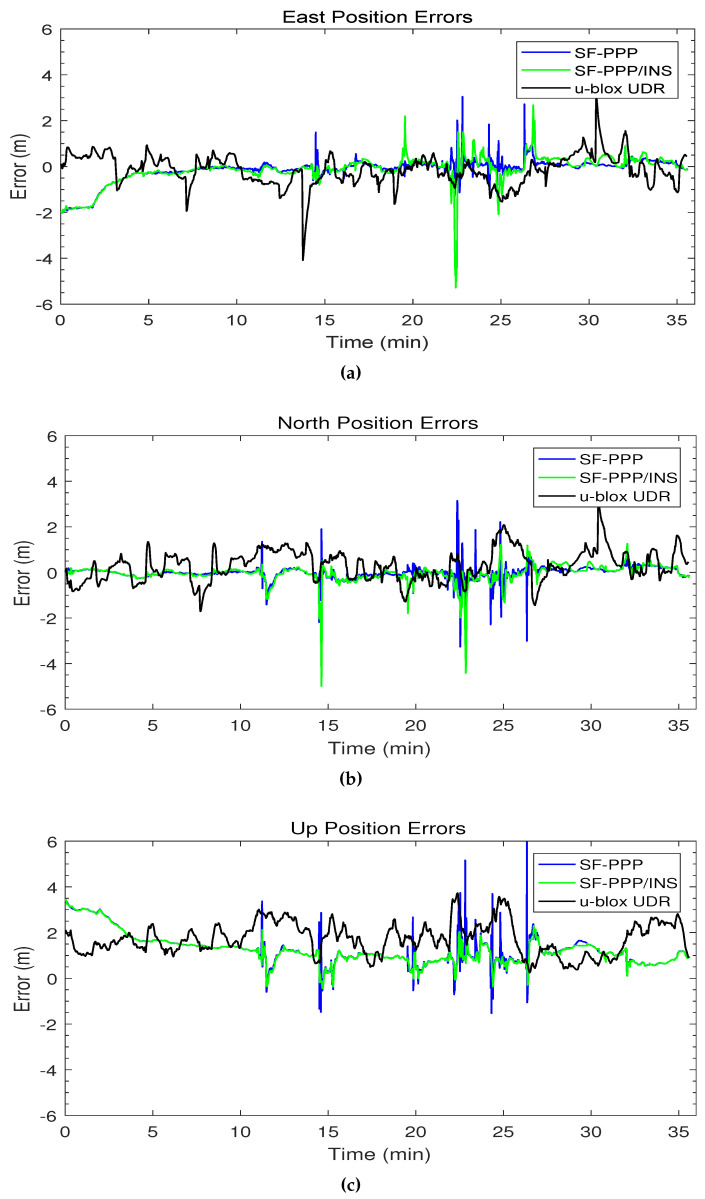
The first trajectory position errors versus time in (**a**) east, (**b**) north, and (**c**) up directions.

Figure 6 shows the 3D position errors for the SF-PPP, the integrated SF-PPP/INS, and the u-blox UDR solutions. The trend of the integrated PPP/INS solution looks similar to the SF-PPP solution; however, the integrated solution smoothed most of the spikes that were in the PPP solution at the epochs with a low number of satellites. The u-blox UDR solution has fewer error spikes than the developed PPP/INS; nevertheless, the u-blox errors have relatively a wider error range compared to the proposed PPP/INS solution after the first 5 min. Both SF-PPP and SF-PPP/INS solutions seem to have a convergence period of 5 min at the beginning of the trajectory that does not exist in the u-blox UDR solution. When this issue was explored further, it was found that, on the day of this test, CNES has recently changed the format of its real-time ionospheric corrections such that it became incompatible with the developed code. Since there were no ionospheric corrections from CNES, the code automatically shifted to use the SBAS ionospheric corrections instead which needed around 5 min to be obtained in real-time.

Table 1 compares the RMS and maximum errors of both u-blox UDR and the developed SF-PPP/INS solutions after the first 5 min to avoid the initial convergence time without ionospheric corrections. The developed SF-PPP/INS system achieved sub-meter RMS horizontal accuracy, and the results were better than the u-blox UDR solution. Furthermore, the RMS error in the vertical direction of the developed system is less than the UDR solution. This error reduction indicates that the integrated solution has benefited from the SF-PPP precise solution in the case of benign GNSS environments.

**Figure 7:**
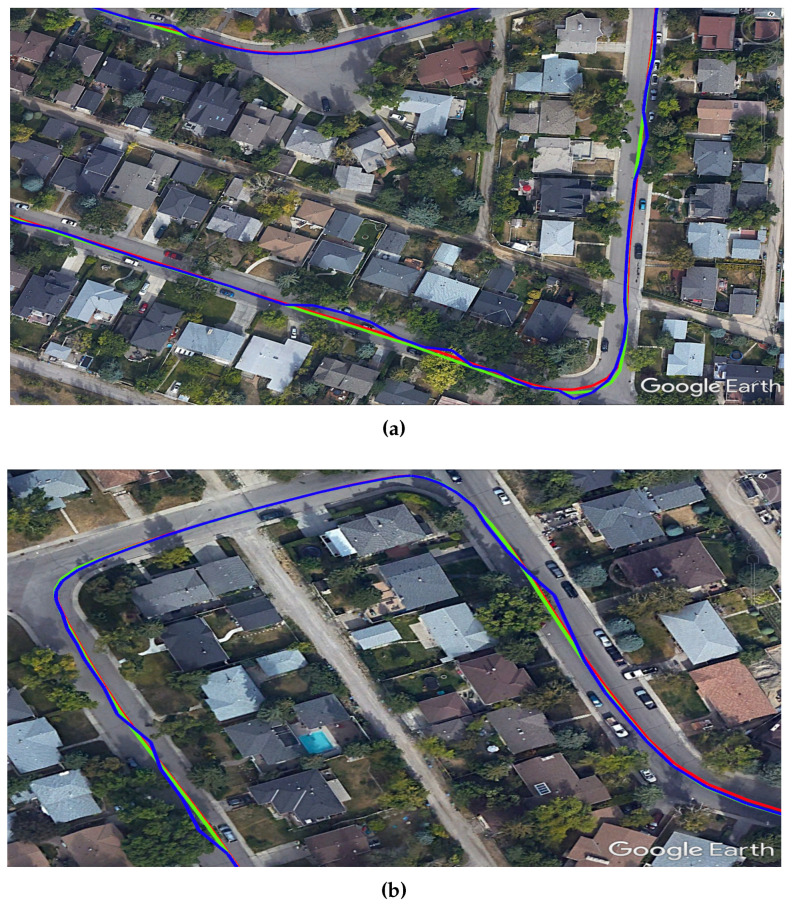
Two examples (**a**) and (**b**) of the developed SF-PPP/INS system performance in suburban areas showing how the system can provide a solution within the driving lane. The three displayed solutions are: reference (red), SF-PPP (blue), and integrated SF-PPP/INS (green).

The final step is to further demonstrate the advantage of our PPP/INS integration in preserving the lane-level accuracy in suburban environments. Figure 7 shows two examples, using Google Earth, of how trees and houses in a suburban environment can affect the PPP solution. It can be seen that the PPP-alone solution (blue) was affected several times by the partial outages of GNSS satellites and this drove the solution outside the driving lane. On the other hand, the integrated PPP/INS solution (green) maintained the lane-level position accuracy, following the reference solution (red) and providing a solution within the driving lane.

### 3.3. Road Test Trajectory 2

**Figure 8:**
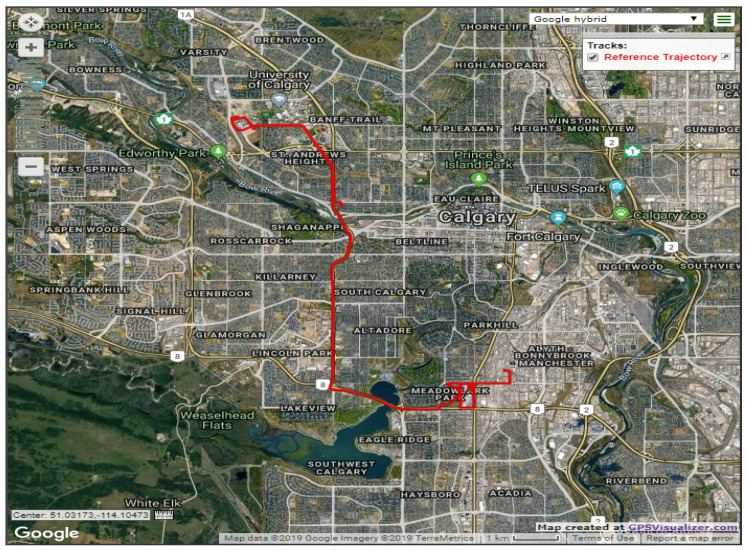
The second test trajectory for SF-PPP/INS integration, Calgary, Alberta, Canada.

**Figure 9:**
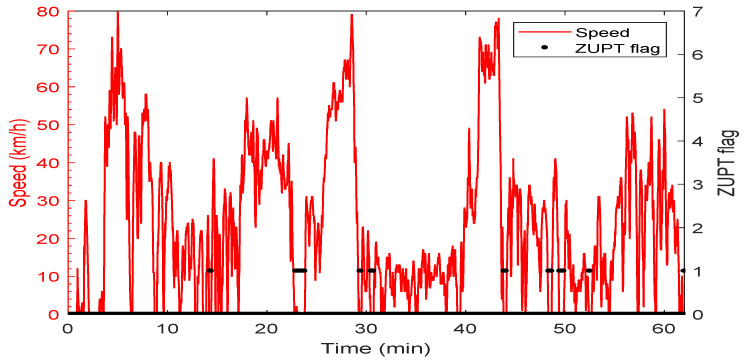
Vehicle speed profile and Zero-Velocity Update (ZUPT) flag of the second trajectory.

**Figure 10:**
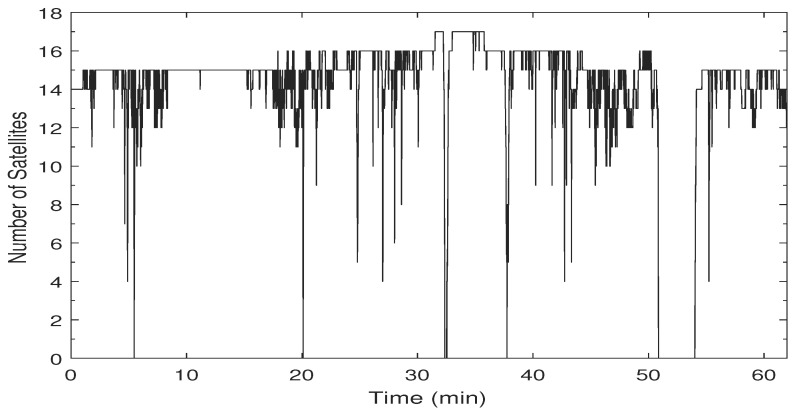
Number of satellites used in the PPP solution of the second trajectory of SF-PPP/INS integration.

The second road test was carried out in Calgary, Alberta, Canada for approximately one hour. Figure 8 shows the trajectory where the car started in a suburban area and moved north to make a few loops in an open-sky environment in the top left part of the trajectory. After that, the car moved back south on a highway inside the city with an 80 km/h speed limit passing under several overpasses and experiencing changing dynamics as can be seen from the speed profile in Figure 9. The last part of the trajectory included underground parking for three minutes, and the test ended in a suburban area. Figure 10 shows the number of satellites used in the PPP solution. The epochs, before the 50th min, at which the number of satellites dropped to five or less correspond mainly to the times when the car moves under an overpass. The long period of zero satellites after the 50th min corresponds to the time when the car went down the underground parking.

Table 2 compares the RMS and maximum position errors of the whole trajectory for both u-blox UDR and the developed SF-PPP/INS solutions. The maximum errors mainly occurred in the underground parking where no PPP solution was available. These results show that the proposed solution has lower errors compared to the u-blox UDR solution in this test.

**Figure 11:**
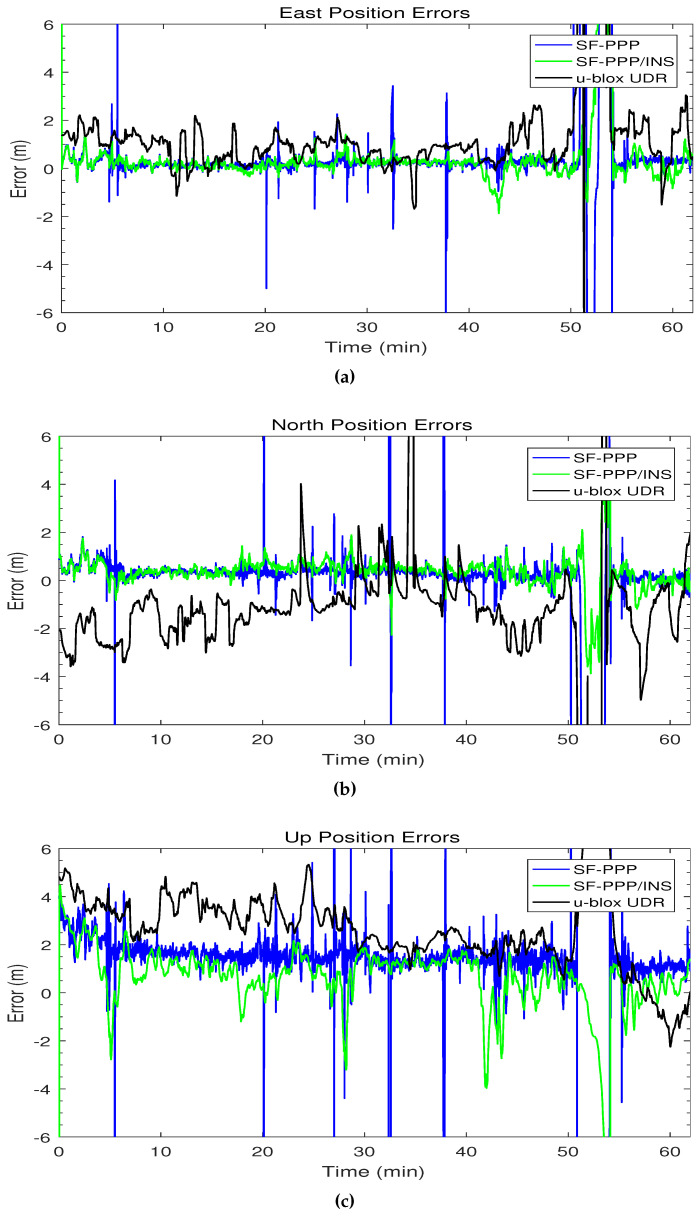
The second trajectory position errors versus time in (**a**) east, (**b**) north, and (**c**) up directions.

Figure 11 shows the 3D position errors within ±6 m for the SF-PPP, the integrated SF-PPP/INS, and the u-blox UDR solutions. The SF-PPP solution suffers from several large error spikes in the east, north, and up directions. Typically, these errors come with a status of “no fix” when the number of satellites is low and the solution is considered unreliable. On the other hand, the developed SF-PPP/INS solution could bridge all the momentary GNSS outages due to overpasses with a sub-meter RMS horizontal error, and provide a continuous solution even in the underground parking. The u-blox UDR position errors are worse than the developed PPP/INS system in most of the trajectory.

For further analysis, the test results are divided into two parts; the first part ends before entering the parking lot and includes the open-sky and highway driving with several overpass bridges. The second part includes driving through underground parking for three minutes. Table 3 shows the position accuracy comparison for the first part of the trajectory. The results show that the PPP/INS solution preserved the sub-meter horizontal accuracy according to the RMS errors compared to a few meters accuracy for u-blox UDR.

**Figure 12:**
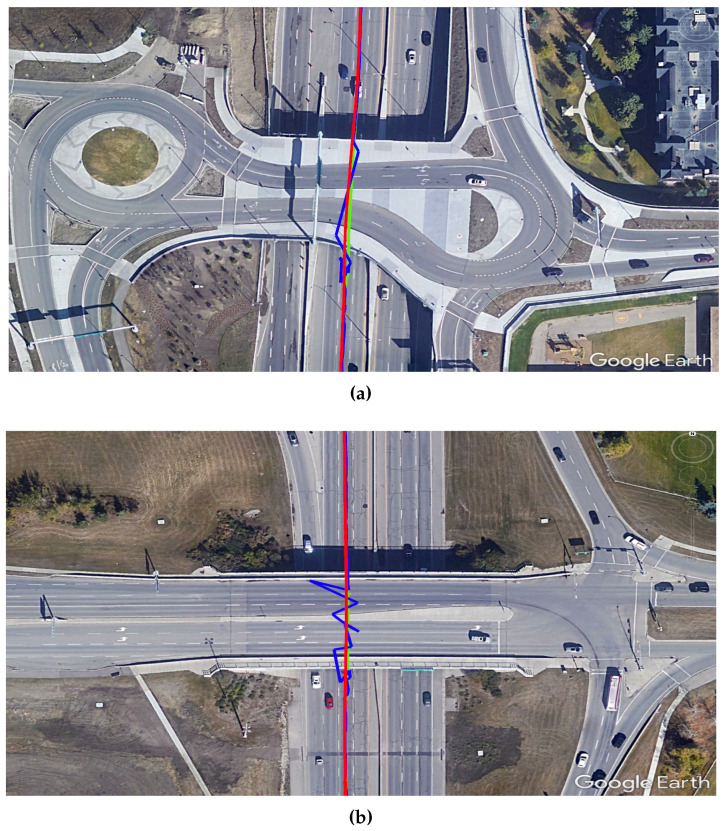
Two examples (**a**) and (**b**) of the developed SF-PPP/INS system performance on highways showing how the system could maintain a solution within the driving lane. The three displayed solutions are: reference (red), SF-PPP (blue), and integrated SF-PPP/INS (green).

Automated vehicles on highways must have a continuous navigation solution with lane-level accuracy. The frequent overpasses impose a challenge on GNSS-based navigation systems including the ones with PPP accuracy. Figure 12 shows two examples of how the developed SF-PPP/INS system could maintain the lane-level accuracy even when the car is moving under a wide overpass.

**Figure 13:**
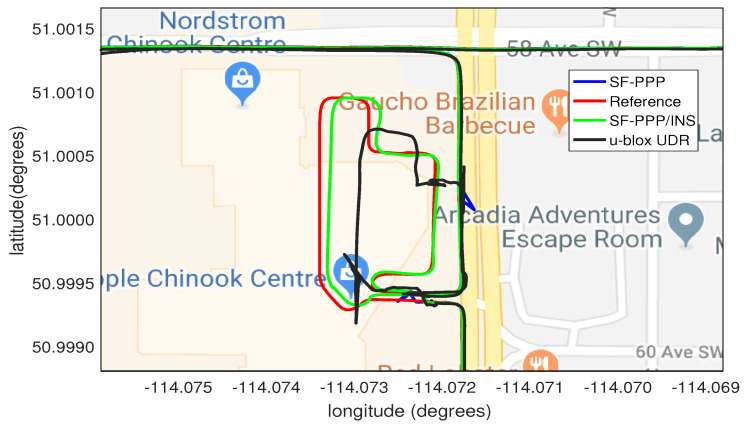
Navigation performance comparison between developed SF-PPP/INS and u-blox UDR in underground parking for 3 min.

In the second part of the trajectory, Figure 13 shows on a Google map how the SF-PPP/INS solution outperforms the u-blox UDR solution in the complete GNSS outage in the underground parking. This is an example of how the developed system behaves in relatively long GNSS outages. Despite the errors reached the meter level, this performance is still acceptable given the utilized low-cost consumer-grade IMU and the long outage period (3 min).

## 4. Discussion

The results in Section 3 have shown that the integrated PPP/INS solution is dominated by the precision of the SF-PPP solution when there is enough number of visible satellites. The INS benefits show up when there is a challenging GNSS environment. When the number of visible satellites is low, typically five or less but can vary based on the satellite geometry and multipath effects, the PPP solution suffers from large spikes. These spikes are accompanied by high covariance and sometimes with no-fix status, which means there is no SF-PPP solution. The integration with INS smooths these spikes and assures the continuity of the navigation solution. The fast convergence of the SF-PPP solution after GNSS outages has contributed to a more stable and reliable integrated solution.

Although the integration with INS helps to reduce the effect of the large errors in the PPP solution, the integrated SF-PPP/INS itself has some error spikes. For example, an error spike occurred in the SF-PPP/INS solution after 22 min in Figure 6a. The fusion of the PPP and INS solutions is based on the quality of the two solutions measured by statistics such as the standard deviation. This is why, in some cases, an inaccurate standard deviation of the PPP solution can mislead the EKF and cause a drift in the integrated solution especially with varying dynamics such as turning. This can be considered as one of the limitations of the loosely-coupled mode of integration.

From the results of the two performed tests, we can also see that the horizontal errors (north and east) are generally less than the vertical errors. One reason is that the vertical DOP (VDOP) is generally higher than the horizontal DOP (HDOP) because we cannot see satellites in the down direction. Another reason is that height errors are strongly affected by tropospheric errors. In our system, the total tropospheric error was a priori modeled as mentioned in Section 2.1. When higher accuracy is required, such as the case with DF-PPP, only the dry component of the tropospheric delay is modeled, whereas the wet component is estimated as an unknown in the EKF.

A crucial factor in the performance of real-time PPP systems, SF or DF, is the availability of the real-time precise corrections. From the results of trajectory 1, when there was an issue with the real-time ionospheric corrections, the solution quality degraded which could result in losing the PPP accuracy. A good practice, which is implemented in the developed system, is to have a backup correction source such as another correction stream or SBAS corrections.

The advantage of using SF-PPP compared to the Standard Point Positioning (SPP), which is the typical solution from low-cost GNSS receivers, was demonstrated by comparing the developed solution with u-blox UDR solution, which is an SPP solution augmented with SBAS corrections. The SF-PPP solution contributed to reducing the horizontal RMS errors of the integrated solution from the meter to sub-meter level of accuracy in suburban environments.

The significance of the presented results is that they represent the performance of the developed system in real challenging situations. The vehicle moved between open-sky and suburban areas passing under several overpasses. Natural GNSS outages, even shorter than simulated outages, are more serious because GNSS signals are gradually re-acquired after the outage. On the contrary, in simulated outages, the signals come back with full strength and a good number of visible satellites right after the introduced outage. The system was also tested in case of a long GNSS outage in underground parking. The good performance during a long GNSS outage is an indication of the well-estimated IMU biases before the outage, which is due to both the PPP accuracy and the reliable estimation approach applied.

The achieved results of the developed PPP/INS system show that this system can play a role in the applications of level 2 autonomy. The system could maintain the lane-level accuracy in suburban areas and on highways. The lane width is typically 3 m or more, e.g., the city of Toronto recommended lane widths between 3 and 4.3 m for the main driving lanes [36], which means that a sub-meter level accuracy is sufficient to keep the vehicle in the lane. The quality of the solution can be increased if higher grades IMU were used; however, this will come at the expense of increasing the system cost.

## 5. Conclusions

The developed system aims at providing a precise real-time low-cost navigation solution for land vehicles with sub-meter accuracy. To fulfill the low-cost requirements, SF-PPP was adopted because it can employ measurements from low-cost SF GNSS receivers in the market. Moreover, low-cost consumer-grade MEMS sensors were utilized for the INS part. The EKF was selected as the integration filter in the developed SF-PPP/INS system. To enhance the performance in the case of GNSS outages, NHC and ZUPT updates were used to limit the solution drift caused by the IMU sensor errors. The performance of the proposed system was investigated using data from two road tests. The testing data included suburban environments, short real GNSS outages, and a 3-min GNSS outage in underground parking. Despite using low-cost IMU and GNSS receivers, the developed PPP/INS system maintained sub-meter level accuracy in suburban areas and during short GNSS outages. Moreover, the system provided a reliable long-term navigation solution in GNSS-denied environments compared to the SPP/SBAS solution provided by u-blox UDR technology. The SF-PPP solution assisted in keeping the positioning solution accuracy to the sub-meter level in suburban environments. Furthermore, fast re-convergence was achieved after the GNSS outages. The INS solution smoothed the PPP output and was utilized to bridge the momentary and long GNSS outages reliably. The developed PPP/INS system is anticipated to play an important role in low-cost automotive applications. It can be utilized in providing sub-meter level accuracy for navigation along highways, which is presently desirable by car manufacturers for their autonomous level 2 operation.

## Figures and Tables

**Table 1:** The first trajectory RMS and maximum position errors after 5 min. The first five minutes were excluded to avoid the effect of the time needed to acquire the ionospheric corrections on the comparison.

	Developed SF-PPP/INS		u-blox UDR	
	RMS Error (m)	MAX Error (m)	RMS Error (m)	MAX Error (m)
Horizontal	0.6	5.4	1.0	4.8
Vertical	1.1	3.4	1.9	3.7

**Table 2:** The RMS and maximum position errors for the whole of the second trajectory. The developed SF-PPP/INS system errors are less than the UDR errors. The maximum errors occurred when the vehicle entered the underground parking.

	Developed SF-PPP/INS		u-blox UDR	
	RMS Error (m)	MAX Error (m)	RMS Error (m)	MAX Error (m)
Horizontal	1.5	12.5	8.8	60.8
Vertical	1.5	8.5	3.3	11.9

**Table 3:** The RMS and maximum position errors, before entering the underground parking in the second trajectory. The developed SF-PPP/INS system maintained sub-meter horizontal accuracy and meter-level vertical accuracy, and was better than UDR solution.

	Developed SF-PPP/INS		u-blox UDR	
	RMS Error (m)	MAX Error (m)	RMS Error (m)	MAX Error (m)
Horizontal	0.7	2.6	2.6	21.1
Vertical	1.4	4.6	2.8	5.3

## Abbreviations

The following abbreviations are used in this manuscript:

CNES	Centre National d’Etudes Spatiales
DCB	Differential Code Bias
DF	Dual-Frequency
DOP	Dilution of Precision
ECEF	Earth-Centered Earth-Fixed
EKF	Extended Kalman Filter
GLONASS	Global Navigation Satellite System
GNSS	Global Navigation Satellite System
GPS	Global Positioning System
IGS	International GNSS Service
IMU	Inertial Measurement Unit
INS	Inertial Navigation System
ISB	Inter-System Bias
LC	Loosely-Coupled
LLF	Local-Level Frame
MEMS	Micro-Electro-Mechanical Systems
NHC	Non-Holonomic Constraints
PPP	Precise Point Positioning
RMS	Root Mean Square
RTS	Real-Time Service
SAE	Society of Automotive Engineers
SBAS	Satellite-Based Augmentation System
SF	Single-Frequency
SPP	Standard Point Positioning
UDR	Untethered Dead Reckoning
ZUPT	Zero-Velocity Update
