# Correction: Elsheikh et al. Low-Cost Real-Time PPP/INS Integration for Automated Land Vehicles. *Sensors* 2019, *19*, 4896

**DOI:** 10.3390/s22155568

**Published:** 2022-07-26

**Authors:** Mohamed Elsheikh, Walid Abdelfatah, Aboelmagd Noureldin, Umar Iqbal, Michael Korenberg

**Affiliations:** 1 Electrical and Computer Engineering Department, Queen’s University, Kingston, ON K7L 3N6, Canada; nourelda@queensu.ca (A.N.); korenber@queensu.ca (M.K.); 2 Electronics and Electrical Communication Engineering Department, Tanta University, Tanta 31512, Egypt; 3 Profound Positioning Inc., Calgary, AB T2P 3G3, Canada; wabdelfatah@profoundpositioning.com; 4 Electrical and Computer Engineering Department, Royal Military College of Canada, Kingston, ON K7K 7B4, Canada; 5 Electrical and Computer Engineering Department, Mississippi State University, Starkville, MS 39762, USA; umar@ece.msstate.edu

The authors wish to make the following corrections in the original paper [1].

The affiliation, Electrical and Computer Engineering Department, Royal Military College of Canada, Kingston, ON K7K 7B4, Canada, has been added to the third author Aboelmagd Noureldin. Consequently, all affiliation information has been updated from the following:

1. Electrical and Computer Engineering Department, Queen’s University, Kingston, ON K7L 3N6, Canada
2. Electronics and Electrical Communication Engineering Department, Tanta University, Tanta 31512, Egypt
3. Profound Positioning Inc., Calgary, AB T2P 3G3, Canada
4. Electrical and Computer Engineering Department, Mississippi State University, Starkville, MS 39762, USA

to

1. Electrical and Computer Engineering Department, Queen’s University, Kingston, ON K7L 3N6, Canada
2. Electronics and Electrical Communication Engineering Department, Tanta University, Tanta 31512, Egypt
3. Profound Positioning Inc., Calgary, AB T2P 3G3, Canada
4. Electrical and Computer Engineering Department, Royal Military College of Canada, Kingston, ON K7K 7B4, Canada
5. Electrical and Computer Engineering Department, Mississippi State University, Starkville, MS 39762, USA

The authors apologize for any inconvenience caused and state that the scientific conclusions are unaffected. The original article has been updated.
